# Reduction of interictal epileptic burden by pulsatile corticoid therapy in children with drug‐resistant epilepsy—How stable is the effect?

**DOI:** 10.1002/epd2.70036

**Published:** 2025-05-10

**Authors:** Katharina Schiller, Tamir Avigdor, John Thomas, Daniel Mansilla, Chifaou Abdallah, Aline Kortas, Gabriele Unterholzner, Raluca Pana, Markus Rauchenzauner, Birgit Frauscher

**Affiliations:** ^1^ Department of Neurology and Neurosurgery Montreal Neurological Hospital and Institute Montreal Quebec Canada; ^2^ Department of Pediatric Neurology Hospital Kaufbeuren Kaufbeuren Bavaria Germany; ^3^ Department of Pediatric Neurology University Hospital Augsburg Augsburg Bavaria Germany; ^4^ Department of Biomedical Engineering Duke Pratt School of Engineering Durham North Carolina USA; ^5^ Department of Pediatrics Medical University Innsbruck Innsbruck Austria; ^6^ Department of Neurology Duke University Medical Center Durham North Carolina USA

**Keywords:** corticosteroids, electroencephalogram, epileptic spike, follow‐up, sleep

## Abstract

**Objective:**

The systematic use of pulsatile corticosteroid therapy (PCT) in children with drug‐resistant epilepsy has been shown to reduce epileptic activity. However, it remains unclear how long this effect will last. The objective of this study was therefore to evaluate the stability of the effect of PCT by assessing the interictal epileptic activity burden (% of electroencephalography (EEG) with interictal epileptic activity, IEA) one year after the end of treatment.

**Methods:**

The final study cohort consisted of 20 children (9 females; mean age 7.6 ± 3.5 years) with various drug‐resistant surgically nonremediable epilepsies who underwent systematic treatment with PCT (20 mg/m^2^ body surface per treatment cycle). EEG recordings during sleep and wakefulness were obtained at baseline, after PCT, and at one‐year follow‐up. IEA burden and sleep spindles (rate per minute) to evaluate sleep physiology were compared between the different time points.

**Results:**

IEA burden was significantly reduced after PCT treatment (4.9% [2.4–20.4] vs. .9% [.2–5.5], *p* = .005, *d* = −.47) and this effect continued to persist at follow‐up (.9% [.2–5.5] vs. 2.7% [.2–7.4], *p* = .99, *d* = .02). At time of follow‐up, 33.3% patients showed a relapse defined by an increase in IEA burden after an initial decrease of at least 50% through PCT. Fast spindle rate (12–16 Hz) tended to be higher after PCT (1.0 ± .8 vs. 1.6 ± .8, *p* = .08, *d* = .59) and remained stable between the end of PCT and follow‐up (1.6 ± .8 vs. 1.6 ± .4, *p* = .98, *d* = .01).

**Significance:**

Our findings suggest that in the majority of patients, PCT led to long‐lasting benefits not only by reducing epileptic activity but also by improving sleep, important for cognitive functions.

**Key points**

- Treatment with corticosteroids can potentially reduce epileptic activity in childhood epilepsies. However, it remains unknown how long this effect will last.
- We evaluated the stability of the positive effect of pulsatile corticosteroid therapy on epileptic activity and sleep in children with drug‐resistant epilepsy.
- The decrease of the interictal epileptic activity burden and the improvement of sleep did not change significantly between ending the treatment and follow‐up.
- Pulsatile corticosteroid therapy may therefore benefit patients with drug‐resistant epilepsy long‐term by reducing epileptic activity and by improving sleep.

## 1 INTRODUCTION

Adrenocorticotropic hormones (ACTH) and corticosteroids are primary treatment options in children with infantile spasms. In addition, corticosteroids have been reported to have a beneficial effect in children with drug‐resistant epilepsies other than West Syndrome.^1^ In a recent meta‐analysis, the use of steroids, mainly orally administered, and the administration of ACTH, was found to reduce seizure frequency and improve the electroencephalogram (EEG) by decreasing interictal epileptic activity determined by subjective evaluation.^2^ In our previous study, we used an automated approach for an objective assessment of interictal epileptic activity to evaluate a standardized treatment with pulsatile corticosteroid therapy (PCT) in patients with drug‐resistant surgically nonremediable epilepsies.^3^ PCT led to reduced interictal epileptic activity in the EEG and a reduced seizure frequency with improved sleep physiology measured by sleep spindle activity.^3^ However, a few long‐term outcome data suggest that there is a risk for relapse of up to 40% in patients using oral corticosteroids or ACTH at follow‐up between three and 12 months following the end of treatment.^2^ Yet, most studies were quite heterogeneous, a baseline was often lacking, and assessment was mainly subjective.^2^ A study of a systematic assessment of EEG changes is missing, especially after a standardized treatment with intravenous administration. In this study, we evaluated the long‐term stability of the reduction of epileptic activity achieved by standardized PCT in children with drug‐resistant epilepsy using a follow‐up measurement after one year. Secondary endpoints were the improvement of sleep as approximated by the rates of sleep spindles and clinical impression at the time of follow‐up.

## 2 MATERIALS AND METHODS

### 2.1 Patient selection

**FIGURE 1:**
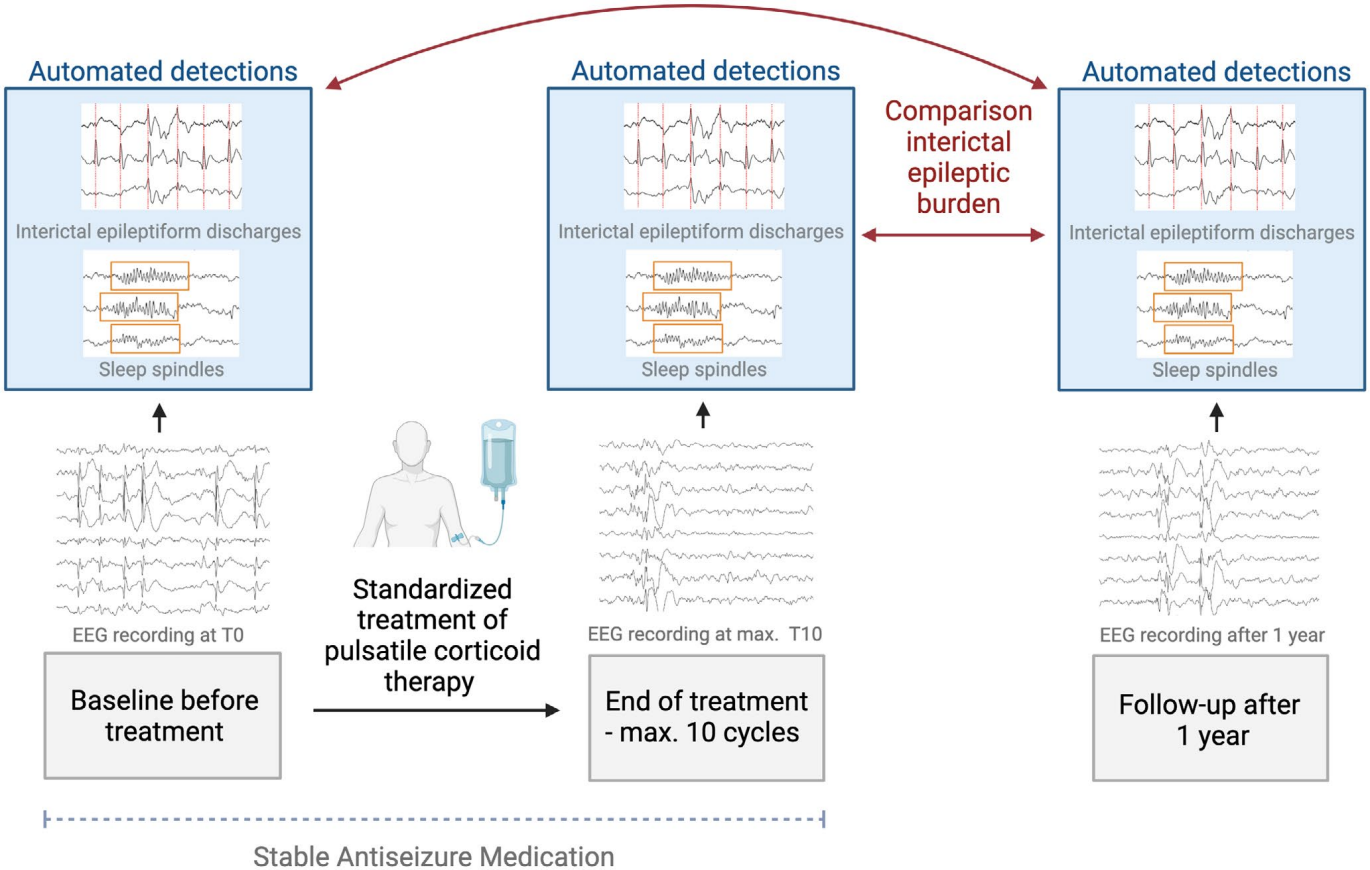
Study design: EEGs were obtained at the baseline before starting PCT, at the end of PCT and at time of follow‐up after one year. EEGs were sleep scored and IEDs as well as sleep spindles were extracted at different time points. Finally, the IEA burden and sleep spindle rates were compared between the baseline, after PCT and at follow‐up to evaluate the stability of the effect of reduced epileptic activity after PCT. This figure was created using Biorender.com.

All consecutive pediatric patients with genetic or suspected genetic drug‐resistant surgically nonremediable epilepsies^4^ who (i) received the standardized protocol of PCT at the Children's Hospital Kaufbeuren and (ii) had a follow‐up after at least one year available, were included in this study. During PCT, patients underwent a standardized procedure of intravenous administration of dexamethasone over three days with a dose of 20 mg/m^2^ body surface for up to 10 cycles.^5^ For futher information, see Schiller et al.^3^ The follow‐up was 1.2 ± .3 years after the end of PCT. While the antiseizure medication (ASM) was kept stable during PCT, ASM could have changed between end of PCT and follow‐up. To account for this, we calculated a standardized ASM load^6^ at the time of PCT and follow‐up (Table 1). The study design is shown in Figure 1. The study was approved by the Ethics board of the Hospital Group Ostallgaeu‐Kaufbeuren (20212).

**TABLE 1:** Clinical data of the study population.

No.	Age (years) at FU; sex	ILAE epilepsy syndrome		Primary indication for PCT	ASM load/day at PCT	ASM load/day at FU	Other therapies between end of PCT and FU	Clinical impression
1	6.4; M	Myoclonic epilepsy in Infancy	Generalized	Increase in seizures	20.0	18.6	–	Increase in seizures 10 months after end of PCT
2	10.2; F	Self‐Limited Epilepsy with Centro‐Temporal Spikes	Focal	Worsening of EEG and increase in seizures	6.4	8.1	–	Stable
3	4.6; F	Myoclonic epilepsy in Infancy	Generalized	Worsening of EEG and increase in seizures	11.6	13.5	–	Stable
4	11.3; F	Self‐Limited Epilepsy with Centro‐Temporal Spikes	Focal	Worsening of EEG and cognitive decline	10.1	0	Ketogenic diet	Stable
5	6.7; M	Myoclonic epilepsy in Infancy	Generalized	Increase in seizures and cognitive decline	11.6	9.9	–	Stable
6	10.8; M	Self‐Limited Epilepsy with Centro‐Temporal Spikes	Focal	Worsening of EEG and cognitive decline	8.4	0	Start of new PCT cycle at FU	Worsening of EEG
7	6.8; M	Epilepsy with Eyelid Myoclonia	Generalized	Worsening of EEG and cognitive decline	1.0	4.9	–	Stable
8	9.6; M	Self‐Limited Epilepsy with Autonomic seizures	Focal	Increase in seizures and cognitive decline	6.8	0	–	Stable
9	2.3; F	Developmental Epileptic Encephalopathy	DEE/PND	Worsening of EEG and increase in seizures	10.0	1.6	–	Increase in seizures
10	7.4; M	Epilepsy with Myoclonic Absences	Focal	Increase in seizures	7.7	10.3	–	Worsening of EEG
11	2.9; M	Developmental Epileptic Encephalopathy	DEE/PND	Worsening of EEG and cognitive decline	0	9.0	–	Stable
12	10.9; M	Self‐Limited Epilepsy with Centro‐Temporal Spikes	Focal	Worsening of EEG and cognitive decline	10.4	7.2	–	Stable
13	10.7; F	Myoclonic Epilepsy in Infancy	Generalized	Worsening of EEG and cognitive decline	7.3	7.7	–	Stable
14	4.4; M	Self‐Limited Epilepsy with Autonomic seizures	Focal	Worsening of EEG and increase in seizures	4.8	4.2	–	Worsening of EEG four months after end of PCT
15	15.1; M	Juvenile Absence Epilepsy	Focal	Worsening of EEG and cognitive decline	1.0	6.0	–	Stable
16	2.0; F	Developmental Epileptic Encephalopathy	DEE/PND	Worsening of EEG and increase in seizures	24.5	16.9	–	Stable
17	4.9; F	Developmental Epileptic Encephalopathy	DEE/PND	Worsening of EEG and increase in seizures	12.7	18.1	–	Stable
18	11.0; F	Epilepsy with Myoclonic Absences	Generalized	Worsening of EEG and increase in seizures	10.3	4.2	–	Stable
19	7.2; F	Myoclonic Epilepsy in Infancy	Focal	Worsening of EEG and increase in seizures	1.7	8.8	–	Stable
20	7.4; M	Self‐Limited Epilepsy with Centro‐Temporal Spikes	Focal	Increase in seizures	4.3	11.8	–	Stable

Abbreviations: ASM, antiseizure medication; DEE, developmental epileptic encephalopathy; F, female; FU, follow‐up; ILAE, International League against Epilepsy; M, male; PCT, pulsatile corticoid therapy; PND, progressive neurological deterioration.

### 2.2 EEGs, sleep scoring, automated detections

EEG recordings with 19 electrodes placed according to the 10–20 system were performed with the Nihon‐Kohden system (Tokyo, Japan) at baseline before PCT, after treatment, and at the time of follow‐up. Sleep scoring was done manually in a bipolar montage in 30‐s epochs.^7^ As in our previous study, a deep learning algorithm was used to determine the interictal epileptic activity burden (% of EEG burdened with interictal epileptic discharges (IEDs) and epileptic bursts, IEA) at the different time points.^8^, ^9^ Sleep spindles (10–16 Hz; .5–3 s; fast spindles (12–16 Hz) and slow spindles (10–12 Hz)) were automatically detected in the common average montage in all 19 channels during a minimum one minute of N2 sleep using a spindle detector validated in epilepsy patients.^10^, ^11^ Detections at the time of an IED were excluded due to potential misdetections. Assessment of spindle rates was possible in 11 patients, with one patient showing abundant epileptic activity at baseline and N2 sleep after PCT and at the time of follow‐up. For each participant, detections were visually cross‐checked by a board‐certified neurophysiologist.

### 2.3 IEA burden

The IEA burden was determined as the percentage duration of EEG burdened with IEDs and bursts of epileptic activity. Each IED was considered 200 ms in duration which corresponds to the maximum duration of graphoelements considered to correspond to a sharp wave. To compare the IEA burden, the most prominent vigilance state in all three EEGs was selected with preference to sleep due to the higher IED rates during NREM sleep.^12^ We defined a patient as relapsed, when IEA burden was reduced for ≥50% after PCT compared to the baseline, and then returned to baseline or higher at time of follow‐up.

Finally, an independent epileptologist, blinded for automated IED markings and the clinical findings, rated the EEGs after PCT and at follow‐up categorizing them as follows: “improvement”, “no change”, or “worsening” regarding the change in epileptic activity.

### 2.4 Evaluation and statistics

Data were tested for normal distribution using the Kolmogorov–Smirnov test. Data are reported as mean ± standard deviation (SD) in case of normally distributed data and median [25th quartile, 75th quartile] otherwise. We computed the Friedman test to compare IEA burden at different time points with subsequently applying the Wilcoxon signed rank test for paired samples. Spindle rates were analyzed using a paired *t*‐test. Effect sizes are reported using Cohen's (normally distributed data) or Cliff's delta (non‐normally distributed data). Statistical analyses were performed using MATLAB 2023 with a *p*‐value < .05 considered to indicate statistical significance.

## 3 RESULTS

### 3.1 Patient demographics

Twenty children (9 females; mean age ± SD at follow‐up: 7.6 ± 3.5 years) were included in the final analysis. According to the International League against Epilepsy (ILAE) classification,^13^ 10 patients had focal epilepsy syndromes, six patients had generalized syndromes and four patients had syndromes with developmental and epileptic encephalopathy with progressive neurological deterioration. MRI was nonlesional in all patients. ASM load did not differ between the time during PCT and at FU (8.5 ± 6.1 vs. 8.0 ± 5.7, *p* = .715, *d* = .08). Clinical information of the study participants is presented in Table 1.

### 3.2 IEA burden

**FIGURE 2:**
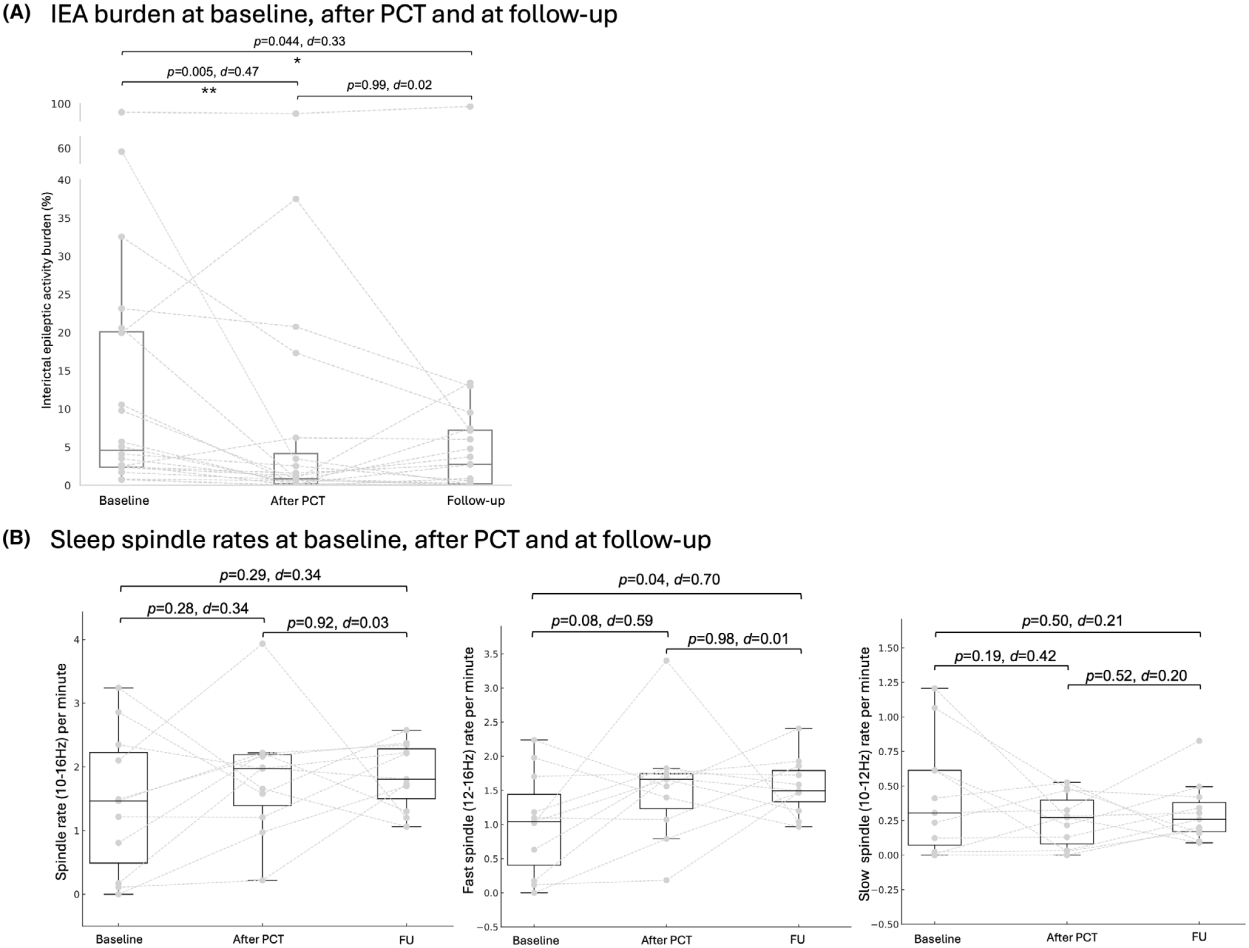
(A) Interictal epileptic activity burden is reduced after PCT compared to baseline and remains unchanged between end of PCT and follow‐up measurement. (B) Sleep spindles (10–16 Hz) did not differ between the different time points (left side). There is a trend of increased fast spindle rates after PCT and a significant improvement at time of follow‐up compared to the baseline (middle). There were no changes in slow spindle rates over time (right).

IEA burden was compared during N2 + N3 in 12 patients, during N1 in six patients, and during wakefulness in two patients. The Friedman test revealed a significant difference in IEA burden between the different time points (*F* = 9.29, *p* = .0096). After PCT, the IEA burden was significantly lower compared to the baseline EEG (Baseline: 4.9% [2.4–20.4] vs. after PCT: .9% [.2–5.5], *p* = .005, *d* = −.47, Figure 2A). This reduction remained stable across the one‐year follow‐up period (after PCT: .9% [.2–5.5] vs. follow‐up: 2.7% [.2–7.4], *p* = .99, *d* = .02). At the time of follow‐up, four patients were free of IEA burden. Four out of 12 patients (33.3%) with an initial decrease of at least 50% after PCT showed a relapse expressed by the return to baseline or higher. According to the clinical information, five patients worsened regarding seizure frequency or EEG pathology between the end of PCT and follow‐up (Table 1).

The independent neurologist rated the EEGs between the end of PCT and follow‐up in four patients with ‘improvement’, in 10 patients with ‘no change’ and in six patients with ‘worsening’. Of the six patients rated with worsening by the epileptologist, five overlapped with the clinical deterioration. Therefore, we observed a high concordance between the automated detections and the ratings of the independent epileptologist.

### 3.3 Sleep spindle rates

The analyses of spindle rates was performed in 11 patients. Fast spindle rates were slightly increased in the EEG after PCT compared to the baseline EEG (Baseline: 1.0 ± .8 vs. after PCT: 1.6 ± .8, *p* = .08, *d* = .59) and the effect persisted between end of PCT and follow‐up (After PCT: 1.6 ± .8 vs. follow‐up: 1.6 ± .4, *p* = .98, *d* = .01) with a significant increase in fast spindle rates at follow‐up compared to baseline (Baseline: 1.0 ± .8 vs. follow‐up: 1.6 ± .4, *p* = .04, *d* = .70). In contrast, all spindle rates (10–16 Hz) and slow spindle rates (10–12 Hz) did not differ between the time points (*p*s > .05, Figure 2B).

## 4 DISCUSSION

Corticosteroids can be effective in reducing epileptic activity in children with drug‐resistant epilepsy. However, it remains unclear if this positive treatment response will persist over time. In this study, follow‐up measurements after one year were objectively analyzed to evaluate the stability of the effect. We found that there were no significant differences in IEA burden between the end of the treatment and the one‐year observational period. More specifically, (i) the reduction in IEA burden persisted between the end of treatment and follow‐up, and (ii), the improvement of sleep expressed by sleep spindles remained stable with a significant improvement compared to the baseline.

The IEA burden was strongly reduced after PCT, which is concordant with previous studies.^2^, ^3^ Then, the burden did not change significantly on a group level between the end of treatment and the follow‐up. At the time of our follow‐up assessment, 33.3% of patients showed a relapse in IEA burden after a decrease of at least 50% reduction achieved through PCT. In the meta‐analysis by Korinthenberg et al.,^2^ the pooled proportion of follow‐up data between three to 12 months revealed a relapse rate of 33% in patients using oral corticosteroids or ACTH. However, only five studies analyzed the relapse of the epileptic activity in the EEG measured by subjective evaluation.^2^ Using an automated approach, we could avoid subjectivity and found a similar relapse rate. Treatment of children with drug‐resistant, surgically nonremediable epilepsies is challenging, and the chance to obtain seizure freedom after multiple ASMs is <5%.^14^ We therefore believe that the observed EEG improvement and seizure reduction through PCT, which persisted in the majority of patients over time, is notable.

Similarly like the IEA burden, the improvement of sleep expressed by sleep spindles did not change between the end of PCT and the follow‐up. In another study, an improvement of intelligence quotient score was reported after the use of oral steroids.^15^ This may coincide with the spindle increase we found in our cohort as spindles were found to be correlated with intelligence.^16^ Therefore, PCT did not only have a positive effect by reducing epileptic activity but also by improving sleep physiology expressed by sleep spindles.

### 4.1 Strengths and potential limitations

We selected an objective approach by using a deep learning algorithm to determine the IEA burden and to measure sleep while avoiding subjectivity in the outcome evaluation. Furthermore, the one‐year observational period after the end of PCT allowed the analysis of the long‐term development of IEA burden. However, there are some limitations to our study. While ASMs were kept stable during PCT, they could have changed until the follow‐up and potentially influenced the development of the epileptic activity. To account for this potential confounder, we calculated a standardized ASM load, and there was no significant difference between both time points. Furthermore, the EEG recordings were of short duration to ensure compliance of the children with this procedure. A longer recording, such as an overnight polysomnography, would offer the possibility to study sleep in more detail. Finally, future research is needed to perform a standardized cognitive testing to evaluate the development of cognitive functions over the different time points.

## 5 CONCLUSION

Systematic PCT has the potential to achieve in the majority of responders long‐lasting positive effects, not only by reducing epileptic activity but also by improving sleep, important for cognitive functions.

## AUTHOR CONTRIBUTIONS

Conception and design of the study: M.R., K.S., B.F.; Acquisition and annotation of data: M.R., A.K., G.U., K.S., B.F., D.M., C.A.; Statistical analysis and interpretation of results: K.S., J.T., T.A., M.R., B.F.; Manuscript preparation: K.S., M.R., B.F.; Manuscript revision and approval: all authors.

## CONFLICT OF INTEREST STATEMENT

None of the authors has any conflict of interest to disclose.

**Test yourself**

1. Which dosage was used per treatment cycle for standardized pulsatile corticosteroid therapy?
  - 5 mg/m^2^ body surface
  - 10 mg/m^2^ body surface
  - 15 mg/m^2^ body surface
  - 20 mg/m^2^ body surface
  - 25 mg/m^2^ body surface
2. Why is the improvement of sleep spindle rate after treatment clinically meaningful?
  - Sleep spindles appear in all sleep stages
  - Sleep spindles represent less sleep fragmentation
  - Sleep spindles are positively associated with cognitive functions
  - Sleep spindles are a marker for sleep pathology
  - Sleep spindles are increased when epileptic activity is increased
3. How high was the relapse rate after one year?
  - 33.3%
  - 10%
  - 66.6%
  - 40%
  - 25.5%
*Answers may be found in the* *supporting information*

## Supporting information


Data S1.

## Data Availability

The data that support the findings of this study are available on request from the corresponding author. The data are not publicly available due to privacy or ethical restrictions.
