# Supplementary material for: Reduction of interictal epileptic burden by pulsatile corticoid therapy in children with drug‐resistant epilepsy—How stable is the effect?
Source: Epileptic Disord. 2025 May 10;27(4):620–8. doi: 10.1002/epd2.70036 (PMC12398194; doi:10.1002/epd2.70036)
Supplement: Supplementary file 1 — Data S1. [file EPD2-27-620-s001.docx]

Answers

1. Correct Answer: D; Patient underwent max. 10 cycles with 20 mg/m^2^ body surface.

2. Correct Answer: C; Sleep spindles were found to be positively correlated with cognitive functions. An improvement in sleep spindle rates may therefore have a beneficial impact on cognition.

3. Correct Answer: A; The relapse rate for patients who had an initial improvement of IEA of $\geq$ 50% after PCT compared to the baseline, and then returned to baseline or higher at time of follow-up, was 33.3%.
